# Dahuang-mudanpi decoction mitigates ALI/ARDS pulmonary inflammation via multi-target regulation of HMGB1

**DOI:** 10.3389/fphar.2026.1835869

**Published:** 2026-06-10

**Authors:** Xianjie Chen, Siyan Li, Xinxin Li, Peng Ye, Jun Zhong, Jianping Lv, Wenjun Ding, Ruiting Lin, Jifei Miao, Hui Li, Sen Ye

**Affiliations:** 1 State Key Laboratory of Traditional Chinese Medicine Syndrome, Guangzhou University of Chinese Medicine, Guangzhou, China; 2 Department of Human Anatomy, School of Basic Medical Sciences, Guangzhou University of Chinese Medicine, Guangzhou, China; 3 The Research Center of Basic Integrative Medicine, School of Basic Medical Sciences, Guangzhou University of Chinese Medicine, Guangzhou, China; 4 School of Basic Medical Sciences, Guangzhou Health Science College, Guangzhou, China; 5 Key Laboratory of Tropical Translational Medicine of Ministry of Education, School of Basic Medical Sciences, Hainan Academy of Medical Sciences, Hainan Medical University, Haikou, China; 6 Guangzhou First People’s Hospital, School of Medicine, South China University of Technology, Guangzhou, China; 7 Shenzhen Bao’an Chinese Medicine Hospital, Guangzhou University of Chinese Medicine, Shenzhen, China

**Keywords:** acute lung injury, dahuang-mudanpi decoction, emodin, HMGB1, paeonol

## Abstract

**Background:**

Acute lung injury (ALI) and acute respiratory distress syndrome (ARDS) are severe inflammatory conditions with high mortality and limited treatment options. Targeting pulmonary inflammation is a critical strategy for improving clinical outcomes. This study was performed to investigate the therapeutic effects and underlying mechanisms of Dahuang-Mudanpi Decoction (DMD), specifically focusing on its active ingredients, paeonol (PAE) and emodin (EMO), and their regulatory role in high mobility group box 1 (HMGB1)-mediated inflammation in ALI/ARDS.

**Methods:**

The therapeutic efficacy of DMD was evaluated using a lipopolysaccharide (LPS)-induced ALI mouse model (n = 8 per group for the DMD dose-screening experiment and n = 6 per group for the component-comparison experiment). High-performance liquid chromatography (HPLC) was employed to quantify the active compounds within DMD. The protective effects were comprehensively assessed by analyzing body weight changes, lung wet/dry (W/D) ratios, and histopathological lung injury scores. Additionally, systemic and localized inflammatory responses were evaluated by measuring hematological parameters and the levels of key inflammatory cytokines, including interleukin-1 beta (IL-1β), tumor necrosis factor-alpha (TNF-α), interleukin-6 (IL-6), and HMGB1. Data were analyzed using one-way analysis of variance (ANOVA) or nonparametric tests, as appropriate.

**Results:**

DMD administration significantly mitigated LPS-induced pulmonary inflammation and attenuated lung injury in the mouse model. Mechanistic evaluations revealed distinct, complementary roles for DMD’s active components, with molecular docking providing supportive structural evidence rather than definitive proof of direct molecular binding. Specifically, PAE was predicted to interact with the first nuclear localization signal (NLS1) region of HMGB1 (Vina score: −4.7 kcal/mol), which corresponded to its observed ability to effectively inhibit the nuclear translocation of HMGB1 and reduce its extracellular release. Conversely, EMO was predicted to bind near the receptor-binding region of HMGB1 (Vina score: −6.3 kcal/mol), suppressing the pro-inflammatory function of extracellular HMGB1.

**Conclusion:**

These findings demonstrate the multi-target regulatory effects of DMD in mitigating ALI-associated inflammation, highlighting PAE and EMO as promising therapeutic agents for ALI/ARDS. The mechanistic insights provide novel perspectives on HMGB1-targeted treatments, suggesting that further optimization of this compound combination could yield advanced therapeutic strategies for severe pulmonary inflammation.

## 1 Introduction

Acute lung injury (ALI) and its most severe manifestation, acute respiratory distress syndrome (ARDS), are life-threatening clinical syndromes characterized by the disruption of the alveolar-capillary barrier, overwhelming inflammatory responses, and refractory hypoxemia (Liu et al., 2023; Yu et al., 2024; Ran et al., 2024; Liang et al., 2024; He et al., 2025). Although supportive treatments have improved, the mortality rate of ALI/ARDS still exceeds 40% (Price and Garcia, 2024), which may be due to the lack of effective drug therapies that can prevent the rapid escalation of the cytokine storm. The pathogenesis of ALI involves a complex network of immune dysregulation, within which the late-acting pro-inflammatory mediator, high mobility group box 1 (HMGB1), acts as a pivotal driver of persistent tissue injury (Wang et al., 2025). Unlike early-phase cytokines such as tumor necrosis factor-alpha (TNF-α) and interleukin-1 beta (IL-1β), HMGB1 functions as a damage-associated molecular pattern with a mechanism of action strictly dependent on its subcellular localization (Chen et al., 2022). During lung injury, HMGB1 is actively secreted by immune cells or passively released from necrotic cells, where it interacts with bacterial components such as lipopolysaccharide (LPS) (Yuan et al., 2024) or binds to pattern recognition receptors, including toll-like receptor 4 (TLR4) (Banks et al., 2023; Mun et al., 2024; Murao et al., 2025) and the receptor for advanced glycation end products, also known as RAGE/AGER (Watanabe et al., 2022; Yao et al., 2025). These interactions trigger the production of pro-inflammatory cytokines (Luo et al., 2022; Xie et al., 2024), exacerbating lung inflammation and damage (Rarick et al., 2024; Mao et al., 2026). The HMGB1/AGER and HMGB1/TLR4 pathways have been strongly implicated in ALI/ARDS pathogenesis (Wang J. et al., 2019; Murao et al., 2025). Some studies have explored the potential of glucocorticoids, such as methylprednisolone, to mitigate ALI/ARDS by reducing HMGB1 secretion (Ji et al., 2018; Li et al., 2020; Deng et al., 2022). However, the therapeutic use of glucocorticoids remains controversial due to potential adverse effects and limited efficacy, particularly in the late stages of ALI (Reichardt et al., 2021; Li et al., 2024; Pirracchio et al., 2024). These findings highlight the need for novel therapeutic strategies capable of precisely modulating the dynamic trajectory of HMGB1.

Dahuang-Mudanpi Decoction (DMD) is a traditional Chinese medicine formula with a long history of use in inflammatory disorders in China. Previous studies have reported the anti-inflammatory effects of DMD in several disease models (Luo et al., 2019; Lin et al., 2023; Congcong et al., 2024), and its major bioactive components, paeonol (PAE) and emodin (EMO), have also been implicated in the regulation of inflammation and HMGB1-related pathways (Miao et al., 2020; Miao et al., 2021; Liu et al., 2022; Hui et al., 2023). PAE has been shown to limit HMGB1 nuclear-cytoplasmic translocation and reduce HMGB1-mediated inflammatory responses, whereas EMO has been associated with suppression of inflammatory signaling related to HMGB1 and nuclear factor kappa B. However, several mechanistic questions remain unresolved. The contribution of PAE and EMO to the overall efficacy of DMD in ALI/ARDS has not been quantitatively linked to their abundance in the decoction. Moreover, most previous studies have examined PAE or EMO separately, leaving unclear whether these two components cooperate within DMD to regulate a common inflammatory target. Since HMGB1-driven inflammation involves multiple functional stages, including nuclear export, extracellular release, and receptor-mediated inflammatory activation, it remains unknown whether distinct DMD-derived components regulate different stages of the HMGB1 cascade.

To address these questions, we quantified PAE and EMO in DMD and prepared a component-matched PAE-EMO combination (PEO) as a mechanistic probe. We then employed LPS-induced ALI mouse models, myeloid-specific *Hmgb1* knockout mice, mouse lung epithelial-12 (MLE-12) cell experiments, alongside bioinformatics analysis, and molecular docking to examine whether PAE and EMO exert complementary regulation of HMGB1-mediated inflammation. This study aimed to clarify how DMD attenuates ALI-associated pulmonary inflammation through coordinated regulation of HMGB1 availability and activity, thereby providing a preclinical mechanistic rationale for further development of DMD-derived therapeutic strategies.

## 2 Materials and methods

### 2.1 Drugs and reagents

The DMD formulation consisted of Da-huang (Rheum officinale Baill. wfo-0000404,454, 12.0 g), Mu-dan-pi (Cortex of Paeonia suffruticosa Andrews, wfo-0000404,454, 3.0 g), Tao-ren (Seed of Prunus persica (L.) Batsch, wfo-0001005458, 9.0 g), Dong-gua-ren (Seed of Benincasa hispida Cogn. wfo-0000562,558, 30.0 g), and Mang-Xiao (Mirabilite, 9.0 g). These materials were mixed, soaked in 300 mL of distilled water for 2 h, and then decocted twice for 15 min each time, using 300 mL for the first time and 150 mL for the second time. The extracts were combined and concentrated to a final volume of 60 mL to obtain a crude drug concentration of 1 g/mL. LPS (*Escherichia coli* O111:B4) was purchased from Sigma-Aldrich (Darmstadt, Germany). Enzyme-Linked Immunosorbent Assay (ELISA) kits targeting IL-1β (E-EL-M0037), HMGB1 (E-EL-M06761), and TNF-α (E-EL-M3063) were purchased from Elabscience (Wuhan, China), while the interleukin-6 (IL-6) ELISA kit (RK00008) was purchased from AbClonal (Wuhan, China). Dexamethasone (Dex) was purchased from Solarbio (D8040, Beijing, China). High-performance liquid chromatography (HPLC)-grade PAE standard (B20266, CAS: 552–41-0, purity ≥98%) and EMO standard (B20240, CAS: 518–82-1, purity ≥98%) were purchased from Yuanye (Shanghai, China). The anti-HMGB1 antibody (ab228624) and the anti-TNF-α antibody (ab183218) were purchased from Abcam (Cambridge, UK), and the anti-glyceraldehyde-3-phosphate dehydrogenase (GAPDH) antibody (2118 S) was purchased from Cell Signaling Technology (Boston, United States). The immunohistochemical staining kit was purchased from Boster (Wuhan, China), and hematoxylin-eosin (H&E) staining kit was purchased from Beyotime (Shanghai, China). All reagents were employed following the instructions provided by the manufacturers.

### 2.2 Animal

All animal care and experimental procedures were conducted in accordance with the guidelines of the Animal Ethics Committee of Guangzhou University of Chinese Medicine (Approval Number: 20230222004). Male C57BL/6 mice aged 6 weeks and weighing 18–20 g were obtained from the Animal Experiment Centre of Guangzhou University of Chinese Medicine in Guangzhou, China. *Hmgb1* ^f/f^, and *Lyz2 Cre* mice were purchased from Cyagen Biosciences, Inc. The mice were housed in a specific pathogen-free laboratory animal room maintained at a constant temperature of 22 °C and a relative humidity of 50%–70%. A 12-h light/dark cycle was maintained, and the mice had free access to food and water. Prior to the experiments, the mice were acclimated to the laboratory conditions for 1 week.

### 2.3 Different doses of DMD in the treatment of LPS-induced ALI model

Animals were randomly allocated into experimental groups (n = 8, per group) as follows: The control group received an intraperitoneal administration of normal saline (10 mL/kg). The ALI model group received an intraperitoneal administration of LPS (10 mg/kg) followed by oral administration of normal saline (10 mL/kg) as a placebo. The positive control group received an intraperitoneal administration of LPS (10 mg/kg) followed by oral gavage of Dex (5 mg/kg, 10 mL/kg) for 3 days (every 24 h).

The DMD treatment groups received an intraperitoneal administration of LPS (10 mg/kg) followed by oral gavage of DMD for 3 days (every 24 h). To clarify the dosage, the DMD stock solutions were prepared at concentrations of 0.25, 0.5, and 1.0 g/mL and administered at a constant gavage volume of 10 mL/kg, which corresponded to low-dose DMD (DMD-L), medium-dose DMD (DMD-M), and high-dose DMD (DMD-H) at 2.5, 5.0, and 10.0 g/kg crude drug equivalents, respectively.

The selected dose range was established based on the classical clinical crude herb amount and body surface area-based dose conversion principles, which are widely utilized for interspecies dose translation. Specifically, based on a clinical daily crude herb dosage of 63 g for a 60-kg adult, the human dose is approximately 1.05 g/kg/day. Applying the body surface area conversion factor between humans and mice (FDA, 2005), the estimated mouse equivalent dose is approximately 12.9 g/kg/day. Therefore, the three evaluated doses in this study successfully covered an explorative dose-response range from low to near-equivalent amounts for comprehensive pharmacological evaluation.

### 2.4 Comparison of DMD and its active ingredients in treating LPS-induced ALI model

Animals were randomly divided into six experimental groups (n = 6). The specific dose of LPS (10 mg/kg, intraperitoneally) was selected to establish a robust acute systemic inflammatory ALI model rather than a survival model. This dosage has been well-documented in previous murine studies to induce marked pulmonary inflammation, lung edema, and intense inflammatory cytokine release (Song et al., 2015). The specific interventions for each group were as follows: The model group received an intraperitoneal administration of LPS (10 mg/kg) followed by oral administration of normal saline (10 mL/kg) as a placebo. The DMD group received an intraperitoneal administration of LPS (10 mg/kg) followed by oral gavage of DMD (0.25 g/mL, 10 mL/kg) for 3 days (every 24 h). The PAE group received an intraperitoneal administration of LPS (10 mg/kg) followed by oral gavage of PAE (1.28 μg/mL, 10 mL/kg) for 3 days (every 24 h). The EMO group received an intraperitoneal administration of LPS (10 mg/kg) followed by oral gavage of EMO (1.48 μg/mL, 10 mL/kg) for 3 days (every 24 h). The PEO group received an intraperitoneal administration of LPS (10 mg/kg) followed by oral gavage of PEO (PAE: EMO = 1.28 μg/mL: 1.48 μg/mL, 10 mL/kg) for 3 days (every 24 h).

### 2.5 Establishment of LPS-induced ALI model by conditional *Hmgb1* knockout mice

Conditional *Hmgb1* knockout mice (*Hmgb1* ^f/f,^ ^*Lyz2*^ ^Cre^) and non-knockout control mice (*Hmgb1* ^f/f^) were randomly assigned to experimental groups as follows: The model group received an intraperitoneal administration of LPS (10 mg/kg) followed by oral administration of normal saline (10 mL/kg) as a placebo. The DMD group received an intraperitoneal administration of LPS (10 mg/kg) followed by oral gavage of DMD (0.25 g/mL, 10 mL/kg) for 3 days (every 24 h). The PEO group received an intraperitoneal administration of LPS (10 mg/kg) followed by oral gavage of PEO (PAE: EMO = 1.28 μg/mL: 1.48 μg/mL, 10 mL/kg) for 3 days (every 24 h).

### 2.6 HPLC

HPLC using an LC-16 Shimadzu system (Kyoto, Japan) and a C18 column (4.6 × 250 mm, 5 μm) from Sigma-Aldrich (Darmstadt, Germany) was employed to identify and quantify the concentrations of PAE and EMO in DMD. The mobile phase consisted of acetonitrile (B) and 0.1% phosphoric acid in water (A). The column temperature was maintained at 30 °C, and the injection volume was set at 10 μL. The detection wavelength was fixed at 264 nm, with a flow rate of 1 mL/min. The gradient elution procedure was as follows: from 0 to 3 min, 70%–60% (A); from 3 to 6 min, 60%–50% (A); and from 6 to 40 min, a constant 50% (A).

### 2.7 Lung wet/dry (W/D) ratio

The superior lobe of the right lung was excised and blotted with filter paper to remove surface moisture before being weighed to record the wet weight. The excised lobe was then placed in an oven (70 °C) and dried until a constant weight was achieved, which was recorded as the dry weight. Finally, the lung W/D ratio was determined, providing a critical measure of pulmonary edema by quantifying the water content in the lung tissue.

### 2.8 Bronchoalveolar lavage fluid (BALF) collection

BALF was collected by tracheal instillation of phosphate buffered saline (PBS), centrifuged, and the supernatant stored at −80 °C.

### 2.9 Histologic analysis

Lung tissues were fixed in 4% buffered paraformaldehyde, embedded in paraffin, sectioned, and stained with H&E. The severity of ALI was assessed using the well-established Smith scoring system (Weng et al., 2022). Specifically, four pathological features—pulmonary edema, alveolar/interstitial inflammation, alveolar/interstitial hemorrhage, and atelectasis or hyaline membrane formation—were each graded semi-quantitatively on a scale of 0–four based on the extent of the lesions: 0, absent; 1, <25%; 2, 25%–50%; 3, 50%–75%; and 4, >75%. The total lung injury score was calculated as the sum of these four parameters, yielding a maximum possible score of 16. Five randomly selected non-overlapping fields per sample were evaluated in a blinded manner.

Immunohistochemical staining was performed using an anti-HMGB1 antibody (ab228624, Abcam). HMGB1 expression was quantified using ImageJ based on five randomly selected non-overlapping fields per sample captured under identical microscope settings. The same thresholding parameters were applied across all groups, and mean optical density (OD) was calculated as integrated OD divided by the selected tissue area. Quantification was performed in a blinded manner.

### 2.10 ELISA

The concentrations of key cytokines and proteins, including TNF-α, IL-6, IL-1β, and HMGB1, in BALF and serum were quantitatively measured utilizing specific ELISA kits and follow the program.

### 2.11 Construction of protein-protein interaction (PPI) network and gene ontology (GO) enrichment analysis

The potential target genes of EMO and PAE were obtained from SwissTargetPrediction (http://www.swisstargetprediction.ch) (Ji et al., 2018). Subsequently, a PPI network was constructed using the STRING database (version 12.0, http://cn.string-db.org/). The organism was set to “*Homo sapiens*,” and a moderate confidence score of 0.400 was established as the minimum required interaction score to ensure statistical significance. In the resulting network, nodes represent proteins, while lines indicate the interactions between them. The CytoNCA plugin of Cytoscape 3.10.1 was employed to visualize the PPI network and to highlight genes with high betweenness centrality through proportional area representation. Finally, proteins from the PPI network that interacted with HMGB1 were selected for GO enrichment analysis. This analysis was conducted using R software (version 4.2.1) and the clusterProfiler package (version 4.4.4), specifically tailored for such enrichment studies. The species focus was on *H. sapiens*, ensuring relevance to human biology. The top 10 statistically significant results were presented, offering insights into the biological processes (BP), cellular components (CC), and molecular functions (MF) associated with these proteins in relation to HMGB1.

### 2.12 Docking

The structures of PAE (PubChem ID: 11092) and EMO (PubChem ID: 3220) were retrieved from the NCBI PubChem dataset (https://www.ncbi.nlm.nih.gov/pccompound/). The structure of HMGB1 (GenBank: CAE48262.1) was retrieved from the SWISS-MODEL (2yrq.1. A, https://swissmodel.expasy.org/interactive/xLrUz2/templates/). We utilized CB-Dock2, a web-based blind docking tool (https://cadd.labshare.cn/cb-dock2/php/index.php, accessed on 07 November 2023), for the molecular docking of PAE and EMO with HMGB1. Ethyl pyruvate and glycyrrhizin were also docked with HMGB1 as reference HMGB1-targeting agents for comparison. This method facilitates the identification of potential binding sites and poses without prior knowledge of the active sites (Wu et al., 2025). Upon uploading the ligand (PAE and EMO) and HMGB1 protein structure files to CB-Dock2, the server’s RDKit module processed the ligand structures by adding hydrogens, assigning partial charges, and generating initial 3D conformations. Concurrently, the protein structure was refined by adding missing hydrogen atoms, removing heteroatoms, and excluding co-crystallized water molecules. The CurPocket algorithm within CB-Dock2 was then employed to detect binding pockets on the protein surface. This method identified the five largest binding cavities, along with their respective docking parameters, including grid center coordinates, size, and volume of the docking box. The Discovery Studio was then employed to detect the interactions with the protein and molecules.

### 2.13 MLE-12 cell culture and treatment

MLE-12 cells were obtained from ATCC and cultured in Dulbecco’s modified Eagle medium/nutrient mixture f-12 medium supplemented with insulin-transferrin-selenium, 10 nM hydrocortisone, 10 nM β-estradiol, 2% fetal bovine serum, and 1% penicillin/streptomycin. For *in vitro* inflammatory modeling, cells were stimulated with LPS (1 μg/mL) for 24 h. Simultaneously, cells were treated with PAE (1.28 μg/mL), EMO (1.48 μg/mL), or PEO. To investigate the exact mechanism of HMGB1 nucleocytoplasmic translocation, the specific inhibitors trichostatin A (TSA) and 2-(4-heptylphenethyl)-6-hydroxybenzoic acid (MG-149) were introduced. The concentration of TSA (0.5 μM) was selected based on established alveolar epithelial cell models to effectively promote HMGB1 nuclear export via regulating acetylation (Neagos et al., 2015), effectively testing whether PAE restrains this exportation process. Meanwhile, MG-149 (10 μM) was utilized to selectively inhibit HMGB1 nucleocytoplasmic translocation based on previous standardization.

### 2.14 Immunofluorescence staining

To detect the nucleocytoplasmic translocation of HMGB1, MLE-12 cells were seeded into 24-well plates with glass coverslips and cultured overnight. Once reaching approximately 70% confluence, the cells were subjected to the respective treatments (LPS and drugs). After the treatments, the cells were washed with PBS, fixed with 4% paraformaldehyde for 15 min at room temperature, and permeabilized with 0.1% Triton X-100 for 10 min. After blocking with 5% bovine serum albumin for 1 h, the cells were incubated with an anti-HMGB1 primary antibody (1:200 dilution) overnight at 4 °C. The following day, the cells were washed three times with PBS and incubated with a fluorophore-conjugated secondary antibody (Alexa Fluor 488) for 1 h at room temperature in the dark. Nuclei were counterstained with 4′,6-diamidino-2-phenylindole (DAPI) for 5 min. Finally, the coverslips were mounted on glass slides using an anti-fade mounting medium. Images were captured using a fluorescence microscope, and the fluorescence intensity was analyzed using ImageJ software.

### 2.15 Cell viability assay

The effect of MG-149 on the viability of MLE-12 cells was evaluated using the Cell Counting Kit-8 (CCK-8) assay. MLE-12 cells were seeded into 96-well plates at a density of 5 × 10^3^ cells per well and cultured overnight. Cells were then treated with various concentrations of MG-149 (including the experimental concentration of 10 μM) for 24 h. Subsequently, 10 μL of CCK-8 solution was added to each well, and the plates were incubated in the dark at 37 °C for an additional 2 h. The OD was measured at 450 nm using a microplate reader. Cell viability was calculated as a percentage relative to the untreated control group.

### 2.16 Western blot

Lung tissues were homogenized in RIPA lysis buffer containing protease inhibitors. Equal amounts of protein were separated by sodium dodecyl sulfate-polyacrylamide gel electrophoresis (SDS-PAGE) and transferred onto polyvinylidene fluoride membrane. After blocking, membranes were incubated with primary antibodies against HMGB1 and TNF-α, and GAPDH overnight at 4 °C, followed by horseradish peroxidase-conjugated secondary antibodies. Protein bands were visualized using an enhanced chemiluminescence system and quantified using ImageJ. Target protein expression was normalized to GAPDH.

### 2.17 Statistical analysis

Data analysis was conducted using GraphPad Prism software, version 9.5.1. Statistical evaluation involved the application of analysis of variance (ANOVA) and nonparametric tests to determine the significance of the findings. A *P*-value of less than 0.05 was established as the threshold for statistical significance.

## 3 Results

### 3.1 DMD attenuates LPS-induced ALI in mice

**FIGURE 1:**
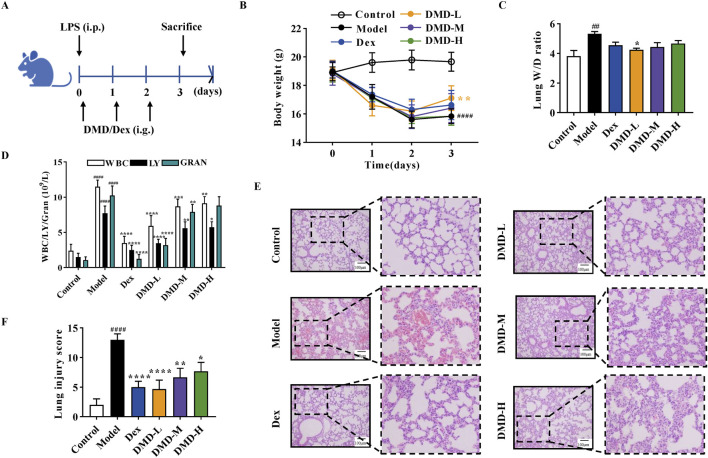
Protective effects of Dahuang-Mudanpi Decoction (DMD) against lipopolysaccharide (LPS)-induced acute lung injury (ALI) in mice **(A)** Experimental design depicting intraperitoneal (i.p.) LPS administration, intragastric (i.g.) DMD or dexamethasone (Dex) treatment, and sample collection timeline **(B)** Body weight changes over time in each group (n = 8) **(C)** Lung wet/dry (W/D) ratio as an indicator of pulmonary edema (n = 4) **(D)** Peripheral blood leukocyte (WBC), lymphocyte (LY), and granulocyte (GRAN) counts across experimental groups (n = 5) **(E)** Representative histological images of lung tissues stained with hematoxylin-eosin (H&E). Black squares indicate magnified regions, showing alveolar architecture and inflammatory infiltration (scale bar: 100 μm, n = 5) **(F)** Quantification of lung injury scores based on histopathological features. Data are presented as mean ± standard error of the mean (SEM). ^##^ *P* < 0.01, ^####^ *P* < 0.0001 vs. control; * *P* < 0.05, ** *P* < 0.01, *** *P* < 0.001, **** *P* < 0.0001 vs. model. DMD-L, low-dose DMD; DMD-M, medium-dose DMD; DMD-H, high-dose DMD.

To assess the protective effect of DMD against ALI, we established a LPS-induced murine model (Figure1A). At 24 h after LPS challenge, mice in the model group showed significant body weight loss, an increased lung W/D ratio, and elevated absolute counts of peripheral leukocytes, lymphocytes, and granulocytes compared with the control group (Figures 1B–D). Treatment with DMD-L attenuated these changes. The effects of DMD-L on pulmonary edema and systemic inflammatory cell counts were comparable to those of Dex. Histopathological examination showed that LPS exposure induced marked lung injury, including alveolar wall thickening, inflammatory cell infiltration, and hemorrhage (Figures 1E,F). In contrast, DMD-L treatment alleviated these pathological changes and improved lung tissue architecture. Lung injury scores were significantly lower in the DMD-L group than in the model group and were comparable to those in the Dex group (Figure 1F). These results indicate that DMD alleviated LPS-induced ALI in mice, as evidenced by reduced pulmonary edema, decreased systemic inflammatory responses, and improved histopathological outcomes.

### 3.2 DMD and PEO attenuate LPS-induced ALI

**FIGURE 2:**
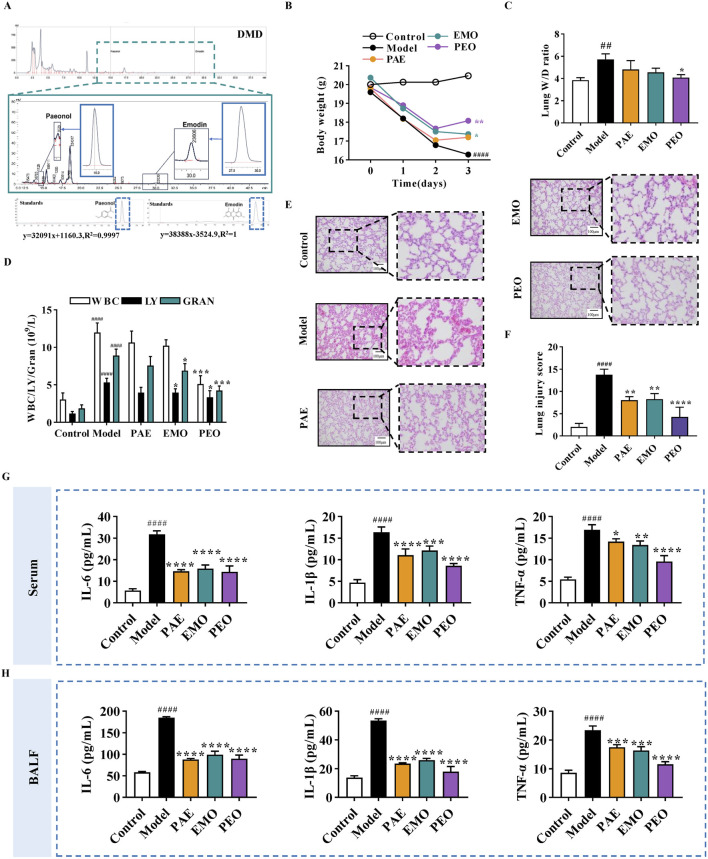
High-performance liquid chromatography (HPLC)-based identification of paeonol (PAE) and emodin (EMO) in Dahuang-Mudanpi Decoction (DMD) and protective effects of PAE, EMO, and PAE-EMO combination (PEO) in lipopolysaccharide (LPS)-induced acute lung injury (ALI) **(A)** Representative HPLC chromatogram of DMD extract (1 g/mL), showing the characteristic peaks of PAE and EMO. HPLC chromatograms of PAE (upper) and EMO (lower) standard solutions (50 μg/mL) **(B)** Body weight changes in each group (n = 6) **(C)** Lung wet/dry (W/D) ratio in each group (n = 3) **(D)** Hematological parameters in each group (n = 5) **(E)** Representative hematoxylin-eosin (H&E)-stained lung sections showing histopathological changes across experimental groups (scale bar: 100 μm, n = 5) **(F)** Quantification of lung injury scores based on histopathological assessment **(G)** Serum levels of interleukin-6 (IL-6), interleukin-1 beta (IL-1β), and tumor necrosis factor-alpha (TNF-α), measured by enzyme-linked immunosorbent assay (ELISA) (n = 3) **(H)** Levels of IL-6, IL-1β, and TNF-α in bronchoalveolar lavage fluid (BALF), determined by ELISA (n = 3). PEO consists of PAE and EMO combined according to their HPLC-quantified concentrations in DMD. Data are means ± standard error of the mean (SEM). ^##^ *P* < 0.01, ^####^ *P* < 0.0001 (compared to control), * *P* < 0.05, ** *P* < 0.01, *** *P* < 0.001, **** *P* < 0.0001 (compared to model group), ^ns^ *P* > 0.05 (compared to model group).

To elucidate the pharmacological basis of DMD in the treatment of ALI, we first used HPLC to quantify its major active constituents (Figure 2A). Chromatographic analysis identified characteristic peaks corresponding to the retention times of PAE and EMO standards. The concentrations of PAE and EMO in the DMD extract were 1.28 μg/mL and 1.48 μg/mL, respectively (Tables 1–3). Based on this ratio, we formulated a PEO to evaluate its contribution to the overall efficacy of the decoction. The PEO showed protective effects similar to those of the complete DMD formulation in preventing weight loss, reducing the lung W/D ratio, and suppressing peripheral leukocytosis (Figures 2B–D). Histological examination showed that both treatments alleviated alveolar congestion, inflammatory cell infiltration, and structural disruption, with significantly lower lung injury scores than those in the model group (Figures 2E,F). In addition, ELISA results showed that DMD and PEO reduced the levels of IL-6, IL-1β, and TNF-α in both serum and BALF (Figures 2G,H). In parallel with the reduction of pro-inflammatory cytokines, Western blot analysis revealed that the LPS-induced upregulation of HMGB1 in lung tissues was markedly suppressed. This inhibitory effect was consistently observed across varying doses of DMD, as well as following treatments with the individual constituents (PAE and EMO) and PEO (Supplementary Figures S1A, B). These results suggest that DMD attenuated LPS-induced ALI and that PEO contributed substantially to its protective effect.

**TABLE 1:** High-performance liquid chromatography (HPLC) measurement of peak areas for different concentrations of paeonol (PAE) standards.

Concentration (μg/mL)	50	10	5	1	0.5
1	1,595,127	307,093	161,778	34,190	29,696
2	1,612,579	298,137	163,439	34,468	33,527
3	1,620,821	297,491	167,218	36,592	30,873
$\bar{x}$	1,609,509.00	300,907.00	164,145.00	35,083.33	31,365.33
SD	10,711.80	4,382.17	2,276.29	1,072.81	1,602.28
RSD	0.006655	0.014563	0.013868	0.030579	0.051084

**TABLE 2:** High-performance liquid chromatography (HPLC) measurement of peak areas for different concentrations of emodin (EMO) standards.

Concentration (μg/mL)	50	10	5	1	0.5
1	1,845,714	374,127	194,652	26,423	15,378
2	1,936,489	379,662	192,153	26,590	15,199
3	1,964,505	385,127	197,470	26,558	14,963
$\bar{x}$	1,915,569.00	379,638.70	194,758.30	26,523.67	15,180.00
SD	50,702.07	4,490.76	2,171.96	72.38	169.95
RSD	0.026468	0.011829	0.011152	0.002729	0.011196

**TABLE 3:** Concentration of paeonol (PAE) and emodin (EMO) in Dahuang-Mudanpi Decoction (DMD).

Group	Curve area of PAE	Concentration of PAE (μg/mL)	Curve area of EMO	Concentration of EMO (μg/mL)
1	42,883	1.300,137	53,954	1.497,314
2	41,822	1.267,075	53,168	1.476,839
3	41,549	1.258,568	52,160	1.450,581
$\bar{x}$	42,084.67	1.27526	53,094	1.474,911
SD	575.4038	0.01793	734.2643	0.019127
RSD	0.013673	0.01406	0.01383	0.012969

### 3.3 Bioinformatics and docking analyses support distinct HMGB1-related regulatory patterns of PAE and EMO

**FIGURE 3:**
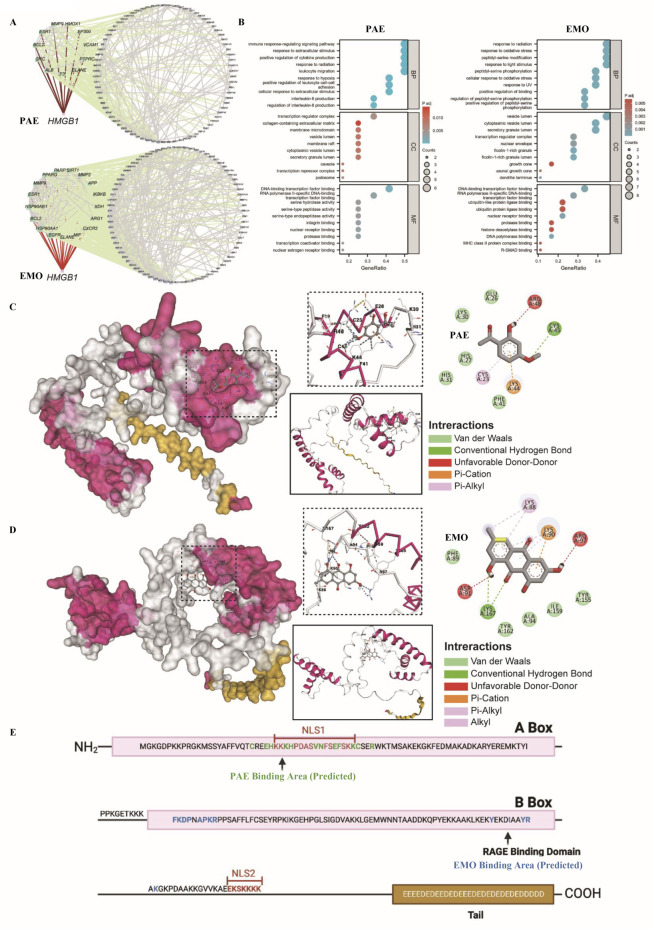
Bioinformatics and molecular docking analysis of paeonol (PAE) and emodin (EMO) in high mobility group box 1 (HMGB1) regulation **(A)** Protein-protein interaction (PPI) networks depicting the interactions between HMGB1 and the predicted target proteins of PAE (upper panel) and EMO (lower panel). Red edges indicate direct interactions with HMGB1, while green edges denote indirect associations **(B)** Gene ontology (GO) enrichment analysis of HMGB1-associated target proteins for PAE (left) and EMO (right), categorized into biological processes (BP), cellular components (CC), and molecular functions (MF) **(C)** Molecular docking simulation of PAE binding to HMGB1, with close-up views of predicted binding residues and intermolecular interactions **(D)** Molecular docking simulation of EMO binding to HMGB1, with close-up views of predicted binding residues and intermolecular interactions. The HMGB1 protein structure is shown in surface representation, with the A Box and B Box domains in pink and the acidic tail region in yellow **(E)** Schematic representation of HMGB1 sequence domains, including A Box, B Box, acidic tail, the first nuclear localization signal (NLS1), the second nuclear localization signal (NLS2), receptor for advanced glycation end products (RAGE)-binding domain, and the predicted binding regions of PAE and EMO.

To explore the potential relationship between PAE, EMO, and HMGB1, candidate targets were predicted using SwissTargetPrediction. A total of 100 predicted targets were identified for each compound (Supplementary Tables S1, S2). PPI network analysis was conducted using STRING and Cytoscape to evaluate interactions between these targets and HMGB1. The results revealed direct associations between HMGB1 and several key proteins, including SRC, BCL2, ESR1, MMP9, HMOX1, EP300, and VCAM1 for PAE, and EGFR, HSP90AA1, BCL2, PPARG, PARP1, SIRT1, and MMP2 for EMO (Figure 3A). To further investigate the biological functions of these interacting proteins, GO enrichment analysis was performed (Figure 3B). In the BP category, PAE-related targets were enriched in pathways involved in immune regulation and responses to extracellular stimuli, whereas EMO-related targets were primarily enriched in oxidative stress responses and vesicle lumen-related functions. In the MF category, PAE-associated targets were enriched in transcription factor binding and nuclear receptor binding, whereas EMO-associated targets were enriched in histone deacetylase binding and ubiquitin-protein ligase binding. These findings suggest that PAE may regulate immune responses through nuclear pathways, whereas EMO may modulate extracellular inflammatory signaling.

Molecular docking simulations were conducted using CB-Dock 2.0 to evaluate the predicted interaction patterns of PAE and EMO with HMGB1 (Figures 3C–E; Table 4). The docking analysis predicted that PAE may interact with the first nuclear localization signal (NLS1)-related region of HMGB1 (Figures 3C,E). This region is associated with HMGB1 nuclear localization and nuclear-cytoplasmic trafficking. The structural model suggested that PAE formed hydrogen bonds and π–π interactions with residues near this region (Table 5). Because HMGB1 nuclear-cytoplasmic trafficking is regulated by post-translational modifications within its nuclear localization regions, this predicted interaction provides structural support consistent with the observed effect of PAE on HMGB1 localization and secretion. However, docking analysis alone cannot determine whether PAE directly affects HMGB1 acetylation or its interaction with nuclear export machinery.

**TABLE 4:** Molecular docking parameters of Dahuang-Mudanpi Decoction (DMD)-derived compounds and reference high mobility group box 1 (HMGB1)-targeting ligands with HMGB1.

Protein receptor	Ligand	Vina score (kcal/mol)	Cavity volume (Å^3^)	Center (x, y, z)	Docking box size (x, y, z)
HMGB1	PAE (Cid 11092)	−4.7	215	25, 27, −21	18, 18, 18
HMGB1	EMO (Cid 3220)	−6.3	393	−6, −11, 0	19, 19, 19
HMGB1	Ethyl pyruvate (CID 12041)	−3.8	313	21, 21, −15	16, 16, 16
HMGB1	Glycyrrhizin (CID 14982)	−6.4	215	25, 27, −21	28, 28, 28

**TABLE 5:** Predicted contact residues of high mobility group box 1 (HMGB1) interacting with paeonol (PAE) or emodin (EMO).

Protein receptor	Molecular ligand	Contact residues
HMGB1	PAE (Cid 11092)	PHE19 CYS23 GLU26 HIS27 LYS30 HIS31 PHE41 LYS44 CYS45 ARG48
HMGB1	EMO (Cid 3220)	LYS88 PHE89 LYS90 ASP91 ALA94 PRO95 ARG97 TYR155 ILE159 TYR162 LYS167

In contrast, EMO showed a more favorable predicted interaction near HMGB1 regions associated with extracellular receptor binding, including the RAGE-related binding region (Figures 3D,E; Table 4). This predicted interaction pattern was consistent with the functional observation that EMO suppressed inflammatory cytokine release without markedly reducing HMGB1 secretion. Thus, the docking results provide supportive structural evidence for the distinct HMGB1-related effects of PAE and EMO, but should not be interpreted as direct proof of molecular binding or domain-specific inhibition.

To place these predicted interactions in context, ethyl pyruvate and glycyrrhizin were included as reference HMGB1-targeting agents in the docking analysis (Supplementary Figures S2A, B **;** Table 4). Ethyl pyruvate, a reported inhibitor of HMGB1 release, showed a predicted Vina score of −3.8 kcal/mol. Glycyrrhizin, a known HMGB1-binding ligand, showed a more favorable predicted Vina score of −6.4 kcal/mol. This result is consistent with the established role of glycyrrhizin as an HMGB1-binding compound. However, docking scores should not be interpreted as direct indicators of biological efficacy. These comparative results were used only to provide a reference context for the predicted interactions of PAE and EMO with HMGB1.

### 3.4 DMD and PEO reduce HMGB1 extranuclear localization and extracellular release

**FIGURE 4:**
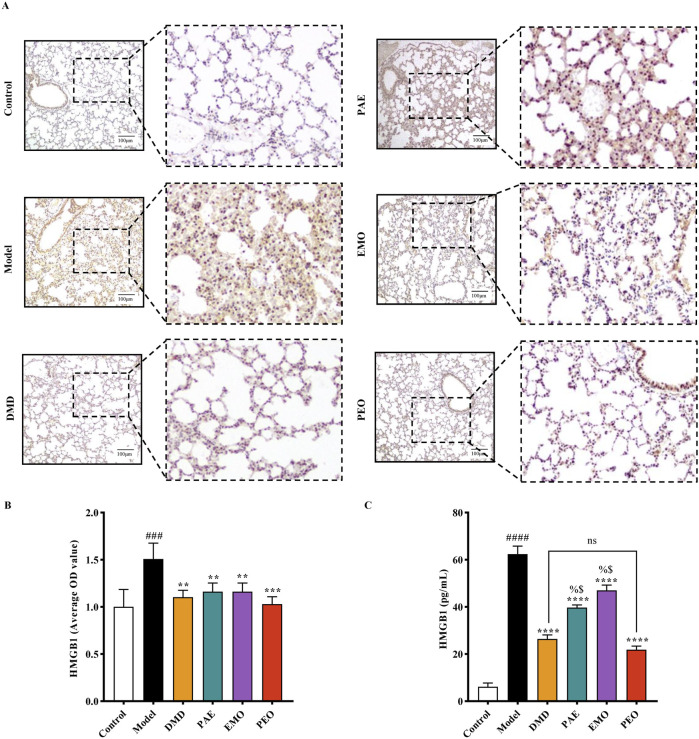
Regulation of high mobility group box 1 (HMGB1) expression by Dahuang-Mudanpi Decoction (DMD), its active ingredients, and PAE-EMO combination (PEO) in acute lung injury (ALI) model **(A)** Representative immunohistochemical staining of HMGB1 in lung tissues from different treatment groups (scale bars: 100 μm and 50 μm, n = 5) **(B)** Quantification of HMGB1 expression based on optical density (OD) values from immunohistochemistry (IHC) analysis **(C)** HMGB1 levels in bronchoalveolar lavage fluid (BALF), measured by enzyme-linked immunosorbent assay (ELISA) (n = 3). Data are presented as means ± standard error of the mean (SEM). ^###^ *P* < 0.001, ^####^ *P* < 0.0001 (vs. control); ^**^ *P* < 0.01, ^***^ *P* < 0.001, ^****^ *P* < 0.0001 (vs. model group); ^%^ *P* < 0.0001 (vs. PEO); ^$^ *P* < 0.0001 (vs. DMD); ^ns^ *P* > 0.05 (not significant). PAE, paeonol; EMO, emodin.

To validate the inhibitory effects of PEO on HMGB1 *in vivo*, we evaluated the localization and release of HMGB1 in lung tissues using immunohistochemistry (IHC) and ELISA. In the ALI model, HMGB1 was predominantly localized in the cytoplasm or extracellular space, consistent with its role in driving inflammatory responses. Treatment with DMD, PAE, EMO, and PEO all reduced HMGB1 levels, although their effects differed mechanistically. PAE treatment notably decreased extracellular HMGB1 but led to nuclear accumulation, suggesting inhibition of nuclear export. In contrast, EMO treatment resulted in an overall reduction in HMGB1 levels without nuclear retention. PEO treatment showed regulatory effects similar to those of DMD, markedly reducing extracellular HMGB1 expression, as confirmed by OD analysis (Figures 4A,B). To further assess HMGB1 secretion into the alveolar space, HMGB1 levels in BALF were quantified using ELISA. PAE significantly reduced HMGB1 secretion, whereas EMO showed a weaker effect than PAE (Figure 4C). Notably, both DMD and PEO effectively suppressed extracellular HMGB1 levels, with no significant difference between them, and both treatments were significantly more effective than PAE or EMO alone. These results indicate that DMD and PEO exert anti-inflammatory effects through coordinated modulation of HMGB1, primarily via PAE-mediated inhibition of nuclear export and EMO-mediated suppression of extracellular HMGB1 activity.

### 3.5 PAE and EMO alleviate inflammation in an LPS-induced *in vitro* model

To investigate whether PAE and EMO exert anti-inflammatory effects by regulating HMGB1 in lung epithelial cells, an LPS-induced inflammatory model was established in the MLE-12 lung epithelial cell line. In addition, TSA was used to promote HMGB1 nuclear export, whereas MG-149 was used to inhibit HMGB1 nucleocytoplasmic translocation, allowing a more precise assessment of how these active ingredients modulate HMGB1. Prior to the mechanistic studies, the effect of MG-149 on MLE-12 cell viability was evaluated to determine an optimal working concentration. As shown in (Supplementary Figure S3A), treatment with MG-149 at 10 μM had no significant impact on cell proliferation or viability compared to the control group. Therefore, this non-toxic concentration was selected to effectively and safely inhibit HMGB1 translocation in subsequent experiments.

**FIGURE 5:**
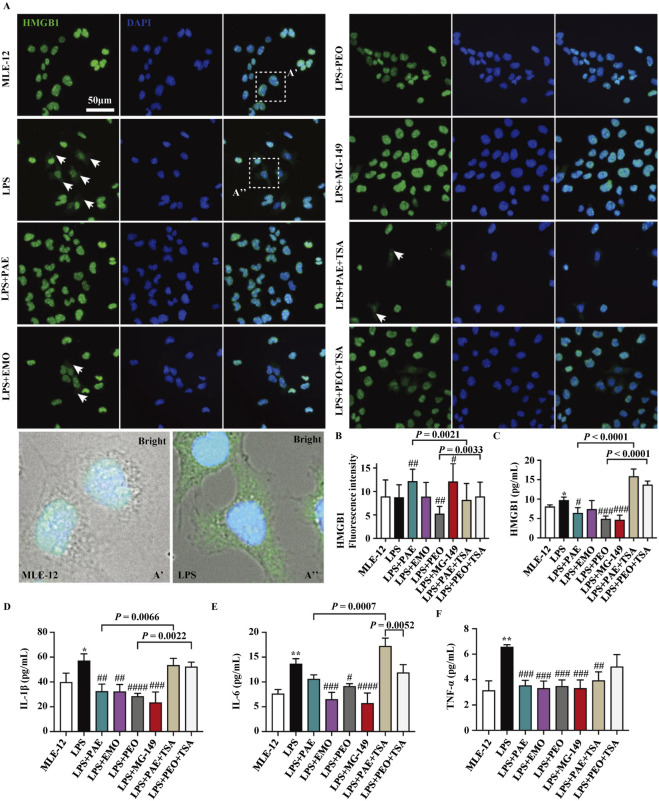
Effects of Dahuang-Mudanpi Decoction (DMD) and its active ingredients on high mobility group box 1 (HMGB1) localization and inflammatory cytokine secretion in a lipopolysaccharide (LPS)-induced *in vitro* model **(A)** Representative immunofluorescence images showing HMGB1 localization (green) in mouse lung epithelial-12 (MLE-12) cells under different treatment conditions (A’ - A″) Magnified views highlighting representative HMGB1 nuclear-cytoplasmic translocation. 4′,6-diamidino-2-phenylindole (DAPI) (blue) indicates nuclei. White arrows highlight cells undergoing HMGB1 nuclear-cytoplasmic translocation (Scale bar: 50 μm) **(B)** Quantification of HMGB1 fluorescence intensity in individual cells (n = 20) **(C)** Levels of secreted HMGB1 in the cell culture supernatant, determined by enzyme-linked immunosorbent assay (ELISA) **(D)** Interleukin-1 beta (IL-1β) **(E)** interleukin-6 (IL-6), and **(F)** tumor necrosis factor-alpha (TNF-α) levels in the cell culture supernatant, determined by ELISA. Data are presented as mean ± standard error of the mean (SEM). * *P* < 0.05, ** *P* < 0.01 (compared to the MLE-12 control group); ^#^ *P* < 0.05, ^##^ *P* < 0.01, ^###^ *P* < 0.001, ^####^ *P* < 0.0001 (compared to the LPS group). PAE, paeonol; EMO, emodin; PEO, PAE-EMO combination; TSA, trichostatin A; MG-149, 2-(4-heptylphenethyl)-6-hydroxybenzoic acid.

Immunofluorescence staining revealed that LPS stimulation induced significant HMGB1 nucleocytoplasmic translocation (Figure 5A’, 5 A″), accompanied by an increase in extracellular HMGB1 levels (Figures 5A–C) and elevated secretion of the pro-inflammatory cytokines IL-6 and TNF-α (Figures 5E– F). EMO treatment did not prevent HMGB1 translocation (Figure 5A) and had no significant effect on intracellular or extracellular HMGB1 levels (Figures 5B,C). However, it effectively reduced the release of inflammatory cytokines, as evidenced by significant decreases in IL-1β, IL-6, and TNF-α levels in the culture supernatant (Figures 5D–F). These results suggest that EMO primarily exerts its anti-inflammatory effects by inhibiting the interaction between extracellular HMGB1 and its downstream pro-inflammatory targets, rather than by directly modulating HMGB1 localization.

In contrast, PAE significantly increased intracellular HMGB1 levels (Figure 5B) while reducing its extracellular secretion (Figure 5C). PAE also markedly suppressed IL-1β and TNF-α secretion (Figures 5D–F). However, when TSA was used to promote HMGB1 nuclear export, the anti-inflammatory effects of PAE were diminished, leading to increased secretion of HMGB1 and IL-6 and thereby exacerbating inflammation. MG-149, which blocks HMGB1 nuclear export, significantly reduced the secretion of HMGB1, IL-1β, IL-6, and TNF-α, demonstrating that nucleocytoplasmic translocation of HMGB1 is a key regulatory step in inflammatory signaling (Figures 5B–F). PEO treatment did not increase intracellular HMGB1 levels but effectively suppressed the secretion of HMGB1, IL-1β, IL-6, and TNF-α (Figures 5B–F). Notably, when TSA was introduced together with PEO treatment, HMGB1 secretion increased, but IL-6 levels remained lower than those observed in the PAE + TSA group. This suggests that even under TSA-induced nuclear export conditions, PEO retains anti-inflammatory activity, which may be primarily attributable to EMO. This finding provides indirect evidence that EMO may attenuate extracellular HMGB1-associated pro-inflammatory signaling, whereas the effect of PAE depends on restricting HMGB1 nucleocytoplasmic translocation. These results indicate that PAE and EMO regulate HMGB1 through distinct mechanisms. PAE suppresses HMGB1 nucleocytoplasmic translocation and extracellular secretion, whereas EMO primarily mitigates inflammation by inhibiting the pro-inflammatory activity of extracellular HMGB1. The complementary actions of these two compounds highlight their potential for combined therapeutic application in inflammatory lung diseases.

### 3.6 Myeloid-specific *Hmgb1* deletion reveals the broad multicellular targeting effects of DMD and PEO

**FIGURE 6:**
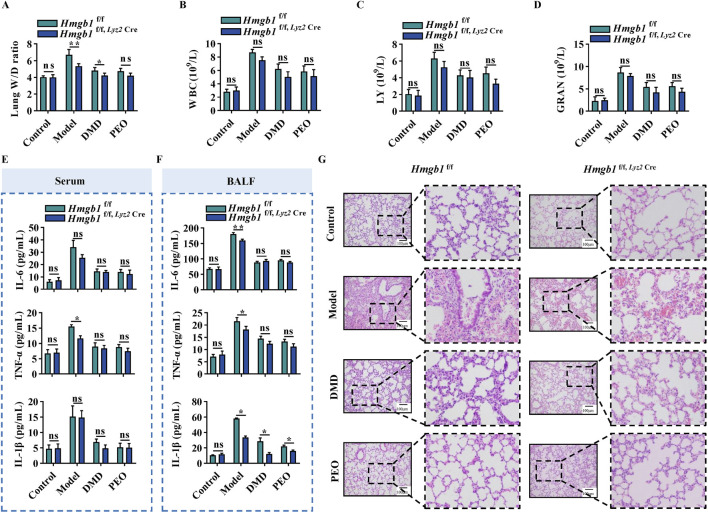
Effects of Dahuang-Mudanpi Decoction (DMD) and PAE-EMO combination (PEO) in conditional myeloid high mobility group box 1 (*Hmgb1*) knockout acute lung injury (ALI) mice. *Hmgb1* ^f/f^ mice were used as non-knockout controls, and *Hmgb1* ^f/f,^ ^*Lyz2*^ ^Cre^ mice were used as myeloid-specific *Hmgb1* knockout mice **(A)** Lung wet/dry (W/D) ratio in each group (n = 5) **(B–D)** Hematological parameters, including **(B)** peripheral blood leukocyte (WBC) **(C)** lymphocyte (LY), and **(D)** granulocyte (GRAN) counts in each group (n = 5) **(E)** Serum levels of interleukin-6 (IL-6), interleukin-1 beta (IL-1β), and tumor necrosis factor-alpha (TNF-α), measured by enzyme-linked immunosorbent assay (ELISA) (n = 4) **(F)** Levels of IL-6, IL-1β, and TNF-α in bronchoalveolar lavage fluid (BALF), determined by ELISA (n = 4) **(G)** Representative hematoxylin-eosin (H&E)-stained lung sections showing histopathological changes across different groups (Scale bar: 100 μm). Data are expressed as means ± standard error of the mean (SEM). * *P* < 0.05, ** *P* < 0.01 between the indicated genotypes (*Hmgb1* ^f/f^ vs. *Hmgb1* ^f/f,^ ^*Lyz2*^ ^Cre^), ^ns^ *P* > 0.05 (not significant). PAE, paeonol; EMO, emodin.

Given that macrophages are an important cellular source of HMGB1, we generated a conditional myeloid-specific *Hmgb1* knockout mouse model to determine whether the therapeutic efficacy of DMD and PEO is strictly dependent on the myeloid compartment. The results showed that deletion of *HMGB1* in myeloid cells partially alleviated pulmonary edema, as indicated by a decrease in the lung W/D ratio compared with that in the model group. However, treatment with DMD or PEO further reduced pulmonary edema, suggesting that their protective effects extend beyond regulation of myeloid-derived HMGB1 (Figure 6A). No significant differences in total leukocyte, lymphocyte, or granulocyte counts were observed between knockout and non-knockout groups (Figures 6B–D). Analysis of serum pro-inflammatory cytokine levels showed that TNF-α levels were significantly reduced in the *Hmgb1* knockout group, whereas IL-6 showed a decreasing trend without statistical significance. Notably, the effects of DMD and PEO on serum cytokine levels did not differ significantly before and after *Hmgb1* knockout, suggesting that their anti-inflammatory properties are not solely dependent on myeloid-derived HMGB1 (Figure 6E). In BALF, levels of IL-6, TNF-α, and IL-1β decreased following myeloid-specific *Hmgb1* knockout. Importantly, IL-1β levels were significantly reduced by DMD or PEO treatment even in knockout mice, further indicating that their therapeutic effects involve mechanisms beyond modulation of myeloid HMGB1 (Figure 6F). To directly evaluate the inflammatory status within the pulmonary microenvironment, lung tissue protein levels of TNF-α were determined by Western blot. In the control mice (*Hmgb1* ^f/f^), LPS induced robust TNF-α protein expression in the lungs, which was remarkably attenuated by DMD and PEO treatments (Supplementary Figures S4A–C). Consistent with our hypothesis, even in the myeloid-specific *Hmgb1* knockout mice (*Hmgb1* ^f/f,^ ^*Lyz2*^ ^Cre^), where lung TNF-α levels were intrinsically lower than those in the *Hmgb1* ^f/f^ model group, treatment with DMD (particularly medium-dose) and PEO still induced a profound and significant reduction in lung tissue TNF-α protein expression. Histopathological examination of lung tissues revealed persistent inflammatory infiltration in the model group despite *Hmgb1* knockout, indicating that myeloid-derived HMGB1 is not the sole contributor to ALI pathogenesis. Treatment with DMD or PEO improved lung tissue morphology, as evidenced by reduced inflammatory cell infiltration and improved alveolar structure, suggesting that additional cellular pathways may contribute to their protective effects (Figure 6G). These results suggest that myeloid-derived HMGB1 contributes more prominently to systemic TNF-α responses, whereas local alveolar IL-1β production remains responsive to DMD/PEO treatment even after myeloid Hmgb1 deletion.

## 4 Discussion

ALI/ARDS remain life-threatening syndromes with limited pharmacological treatment options beyond supportive care (Fadanni and Calixto, 2023; Liu et al., 2025; Hou et al., 2025). Traditional Chinese medicine offers a rich resource for drug discovery, and identifying active compounds with specific molecular targets may aid in developing novel ALI treatments (Liu et al., 2024; He et al., 2021; Wang et al., 2021; Xu et al., 2023; Zhang et al., 2025). In this study, we show that DMD attenuates LPS-induced ALI and that its two quantifiable major constituents, PAE and EMO, contribute to this effect through complementary regulation of HMGB1. Rather than acting through a single inhibitory mechanism, DMD appears to modulate both HMGB1 availability and extracellular HMGB1-driven inflammatory signaling. This provides a mechanistic explanation for why the PEO reproduced much of the anti-inflammatory efficacy of DMD *in vivo*.

HMGB1 is a late-phase inflammatory mediator, actively secreted by immune cells or passively released by damaged cells, amplifying inflammatory cascades in ALI. However, therapeutic targeting of HMGB1 is complex (Wang M. et al., 2019; Jiao et al., 2024). Excessive extracellular HMGB1 promotes inflammatory injury, but complete removal of HMGB1 may be undesirable because of its physiological roles in nuclear homeostasis, tissue repair, and host defense (Chen et al., 2021; Zhao et al., 2021; Al-kuraishy et al., 2022; Ding et al., 2025). Therefore, strategies that modulate HMGB1 activity rather than indiscriminately eliminate HMGB1 may be more suitable for ALI treatment.

Several HMGB1-targeting agents have been reported in inflammatory disease models. Ethyl pyruvate has been shown to inhibit HMGB1 release and protect against lethal systemic inflammation in endotoxemia and sepsis models (Ulloa L et al., 2002). Glycyrrhizin directly binds HMGB1 and inhibits its cytokine activities (Mollica L et al., 2007), whereas thrombomodulin can sequester HMGB1 and prevent HMGB1-RAGE interaction (Abeyama K et al., 2005). These studies support HMGB1 as a feasible therapeutic target in inflammatory diseases. Compared with these relatively defined HMGB1-targeting strategies, the present study suggests that DMD may act through a broader regulatory pattern involving both reduced HMGB1 release and suppression of extracellular HMGB1 activity. However, this interpretation should be considered a working model, because the direct molecular interactions predicted by docking remain to be validated by biochemical binding assays or site-directed mutagenesis. In the comparative docking analysis, glycyrrhizin showed a more favorable predicted Vina score than PAE and EMO, consistent with its known HMGB1-binding property. However, docking affinity alone cannot explain biological efficacy, especially because ethyl pyruvate mainly acts by inhibiting HMGB1 release rather than serving as a classical HMGB1-binding ligand. Therefore, the docking comparison was used only to contextualize the predicted PAE/EMO-HMGB1 interactions.

PAE mainly affected HMGB1 nuclear-cytoplasmic trafficking. Molecular docking analysis predicted that PAE may interact with the nuclear localization signal (NLS)-related region of HMGB1 (Al-kuraishy et al., 2022), which is involved in acetylation-associated nuclear export. Consistent with this prediction, PAE increased intracellular HMGB1 retention, reduced extracellular HMGB1 release, and suppressed inflammatory cytokine secretion in LPS-stimulated MLE-12 cells. When TSA was used to enhance HMGB1 nuclear export, the protective effect of PAE was weakened. Conversely, MG-149 reduced HMGB1 secretion and inflammatory cytokine levels. These results support the notion that restricting HMGB1 nuclear-cytoplasmic trafficking is an important mechanism underlying the anti-inflammatory effect of PAE. Although these findings support a functional relationship between PAE and HMGB1 nuclear-cytoplasmic trafficking, the precise molecular step remains unresolved. Future studies using immunoblotting for acetyl-HMGB1, immunoprecipitation, mutagenesis of NLS-related residues, and assays examining potential export associated with HMGB1 and exportin 1 (XPO1)/chromosome region maintenance 1 (CRM1) will be required to determine whether PAE directly affects HMGB1 post-translational modification or the nuclear export machinery.

In contrast, EMO appears to attenuate the pro-inflammatory activity of extracellular HMGB1, possibly by interfering with receptor-associated HMGB1 signaling. Docking analysis predicted that EMO may interact with HMGB1 regions associated with receptor binding, including interfaces related to AGER (residues 150–183), TLR4 (residues 89–108), and LPS (residues 80–96) (Xu et al., 2018). Functionally, EMO reduced IL-1β, IL-6, and TNF-α release even when HMGB1 secretion was not significantly decreased. Moreover, under TSA-induced enhancement of HMGB1 export, EMO-containing PEO retained the ability to suppress IL-6 more effectively than PAE with TSA. These observations support a model in which EMO mainly attenuates extracellular HMGB1-associated inflammatory signaling rather than preventing HMGB1 release.

The myeloid-specific *Hmgb1* knockout experiment further clarified the cellular boundary of this mechanism. Deletion of *Hmgb1* in myeloid cells partially reduced ALI severity, confirming the contribution of myeloid-derived HMGB1 to inflammatory lung injury. However, DMD and PEO retained anti-inflammatory effects after myeloid *Hmgb1* deletion, indicating that their protective effects are not exclusively dependent on myeloid-derived HMGB1. The different responses of serum TNF-α and BALF IL-1β after myeloid-specific *Hmgb1* deletion may reflect compartment-specific inflammatory regulation. Serum cytokines mainly indicate systemic inflammatory activation, whereas BALF cytokines more directly represent the local alveolar inflammatory microenvironment. The significant reduction in serum TNF-α after myeloid *Hmgb1* deletion suggests that myeloid-derived HMGB1 contributes strongly to systemic TNF-α production. In contrast, the further reduction of BALF IL-1β by DMD or PEO in knockout mice indicates that local pulmonary inflammation was not completely abolished by deletion of myeloid-derived *Hmgb1*. BALF IL-1β may be regulated by additional local mechanisms, including epithelial cell responses, resident macrophage activation, inflammasome-associated signaling, or HMGB1-independent inflammatory pathways. This interpretation is consistent with our MLE-12 cell data and supports the view that DMD-mediated protection is not restricted to myeloid-derived HMGB1.

Taken together, these findings suggest that DMD acts through a dual-stage regulatory pattern: PAE reduces HMGB1 nuclear export and extracellular release, whereas EMO attenuates extracellular HMGB1-associated inflammatory signaling. This complementary mechanism may explain why the PEO reproduced much of the protective effect of DMD and why DMD showed broader anti-inflammatory activity than either monomer alone. Such a mechanism also provides a more precise interpretation of the multi-component nature of DMD: its therapeutic effect may arise not simply from multiple unrelated targets, but from coordinated regulation of different functional stages of a central inflammatory mediator.

Several limitations should be acknowledged. First, DMD-alone and PEO-alone groups were not included in the animal experiments; therefore, the present data do not fully address the baseline biological effects or safety profiles of these treatments under non-inflammatory conditions. Second, molecular docking was used only as a predictive tool. Although the predicted binding patterns were consistent with the functional observations, conventional docking provides static structural predictions and cannot determine the dynamic stability, conformational persistence, or biological relevance of ligand-HMGB1 interactions under physiological conditions. Therefore, future studies should combine molecular dynamics simulations with direct biochemical validation, such as surface plasmon resonance, cellular thermal shift assay, pull-down assay, or site-directed mutagenesis of HMGB1 functional domains, to verify the predicted PAE-HMGB1 and EMO-HMGB1 interactions. Third, TSA and MG-149 were used as pharmacological modulators under LPS-induced inflammatory conditions, but complete TSA-alone and MG-149-alone mechanistic controls would further clarify their basal effects on HMGB1 trafficking. Fourth, DMD is a multi-component herbal formula, and constituents other than PAE and EMO may also contribute to its anti-inflammatory activity. Future studies using comprehensive chemical profiling, component-depletion/add-back experiments, cell-type-specific models, and optimized component-ratio validation will be needed to further refine the proposed HMGB1-centered regulatory model. These limitations do not invalidate the current findings, but define the boundaries of interpretation and indicate the next steps required for mechanistic validation.

## 5 Conclusion

This study demonstrates that DMD and its active ingredients, PAE and EMO, effectively alleviate ALI/ARDS-induced pulmonary inflammation. These effects are achieved through distinct but complementary mechanisms: PAE inhibits HMGB1 nuclear-cytoplasmic translocation and secretion, whereas EMO attenuates the pro-inflammatory activity of extracellular HMGB1. These findings provide a mechanistic basis for the therapeutic effects of DMD and suggest that PAE and EMO could serve as key components in developing optimized treatments for ALI/ARDS.

## Data Availability

The original contributions presented in the study are included in the article/Supplementary Material, further inquiries can be directed to the corresponding authors.
