# Supplementary material for: Dahuang-mudanpi decoction mitigates ALI/ARDS pulmonary inflammation via multi-target regulation of HMGB1
Source: Front Pharmacol. 2026 Jun 10;17:1835869. doi: 10.3389/fphar.2026.1835869 (PMC13291932; doi:10.3389/fphar.2026.1835869)
Supplement: Supplementary file 1 [file DataSheet1.pdf]

Supplementary Material

1 Supplementary Figures and Tables

1.1 Supplementary Figures

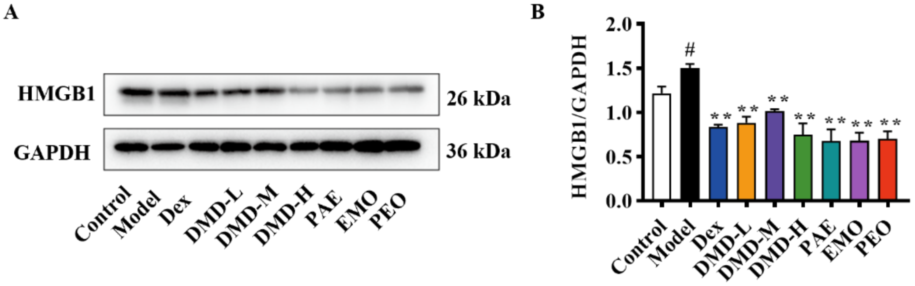

Supplementary Figure S1. Effects of Dahuang-Mudanpi Decoction (DMD), its active ingredients, and paeonol-emodin combination (PEO) on high mobility group box 1 (HMGB1) protein expression in the lung tissues of mice with lipopolysaccharide (LPS)-induced acute lung injury (ALI). (A) Representative Western blot bands showing the expression levels of HMGB1 and the loading control GAPDH in lung tissues from different treatment groups: Control, Model, dexamethasone (Dex), low-dose DMD (DMD-L), medium-dose DMD (DMD-M), high-dose DMD (DMD-H), paeonol (PAE), emodin (EMO), and PEO. (B) Quantitative analysis of the relative protein expression of HMGB1 normalized to glyceraldehyde-3-phosphate dehydrogenase (GAPDH). Data are presented as mean ± standard error of the mean (SEM) (n = 3). $^{\#}$ $P < 0.05$ compared to the control group; ** $P < 0.01$ compared to the model group.

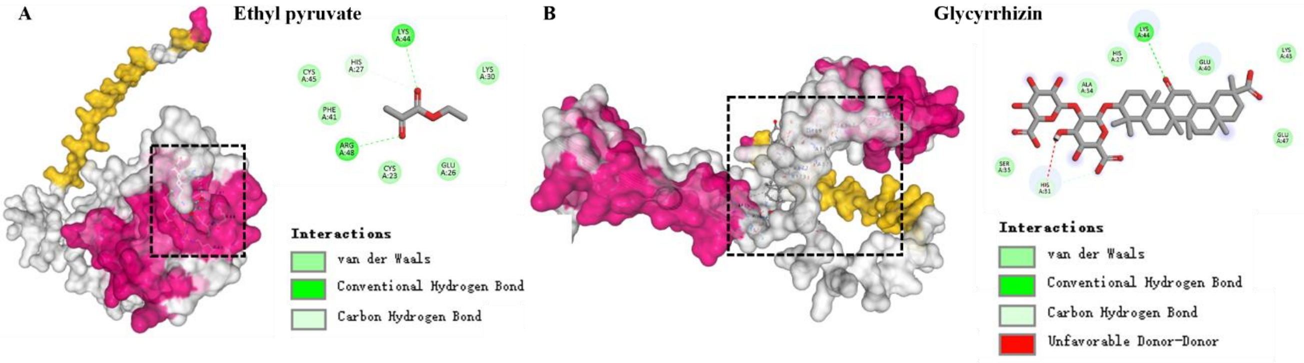

Supplementary Figure S2. Molecular docking simulations of reference high mobility group box 1 (HMGB1)-targeting agents. (A) Molecular docking simulation of ethyl pyruvate binding to HMGB1, with close-up views of predicted binding residues and intermolecular interactions. (B) Molecular docking simulation of glycyrrhizin binding to HMGB1, with close-up views of predicted binding residues and intermolecular interactions. The HMGB1 protein structure is shown in surface representation, with the A Box and B Box domains in pink and the acidic tail region in yellow.

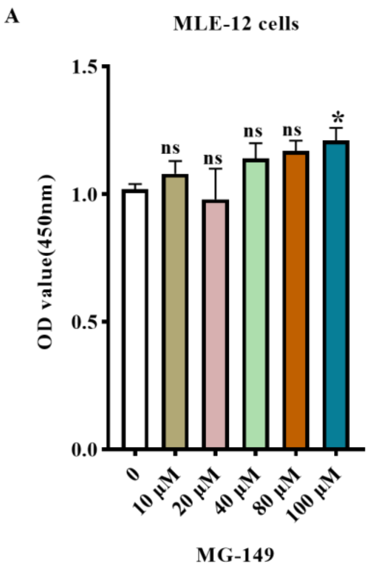

Supplementary Figure S3. Effect of 2-(4-heptylphenethyl)-6-hydroxybenzoic acid (MG-149) on mouse lung epithelial-12 (MLE-12) cell viability. (A) MLE-12 cells were treated with varying concentrations of MG-149 (0, 10, 20, 40, 80, and 100 μM) for 24 h. Cell viability was assessed using the Cell Counting Kit-8 (CCK-8) assay by measuring the optical density (OD) at 450 nm. Data are presented as mean ± standard error of the mean (SEM) (n = 3). ***** *P* < 0.05 compared with the untreated control group (0 μM); ns, not significant compared with the untreated control group.

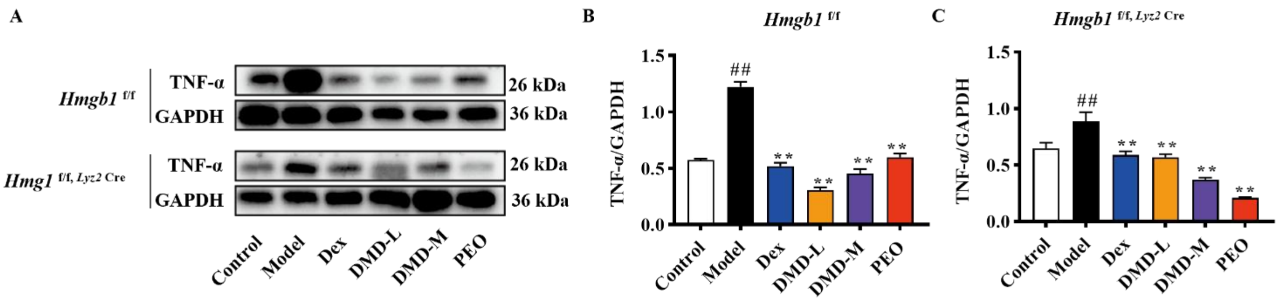

Supplementary Figure S4. Effects of Dahuang-Mudanpi Decoction (DMD) and paeonol-emodin combination (PEO) on tumor necrosis factor-alpha (TNF-α) protein expression in lung tissues of conditional myeloid high mobility group box 1 (*Hmgb1*) knockout (*Hmgb1* $^{\mathrm{f/f},\,\mathit{Lyz2}\ \mathrm{Cre}}$) and control (*Hmgb1* $^{\mathrm{f/f}}$) mice. (A) Representative Western blot images showing the expression of TNF-α and GAPDH in the lung tissues of both mouse genotypes across different experimental groups. (B) Quantitative analysis of the relative TNF-α protein expression normalized to glyceraldehyde-3-phosphate dehydrogenase (GAPDH) in *Hmgb1* $^{\mathrm{f/f}}$ mice. (C) Quantitative analysis of the relative TNF-α protein expression in *Hmgb1* $^{\mathrm{f/f},\,\mathit{Lyz2}\ \mathrm{Cre}}$ mice. Data are presented as mean ± standard error of the mean (SEM) (n = 3). $^{\#\#}$ *P* < 0.01 compared to the corresponding control group; ****** *P* < 0.01 compared to the corresponding model group.

1.2 Supplementary Tables

Table S1 Prediction Targets of Paeonol

Target	Common name	Uniprot ID	ChEMBL ID
Serine/threonine-protein kinase/endoribonuclease IRE1	ERN1	O75460	CHEMBL1163101
HMG-CoA reductase	HMGCR	P04035	CHEMBL402
Carbonic anhydrase IX	CA9	Q16790	CHEMBL3594
Histone acetyltransferase p300	EP300	Q09472	CHEMBL3784
Carbonic anhydrase II	CA2	P00918	CHEMBL205
Carbonic anhydrase I	CA1	P00915	CHEMBL261
Carbonic anhydrase VII	CA7	P43166	CHEMBL2326
Carbonic anhydrase XII	CA12	O43570	CHEMBL3242
Carbonic anhydrase XIV	CA14	Q9ULX7	CHEMBL3510
Monoamine oxidase B	MAOB	P27338	CHEMBL2039
Fatty acid synthase	FASN	P49327	CHEMBL4158
CDGSH iron-sulfur domain-containing protein 1	CISD1	Q9NZ45	CHEMBL1795168
Alkaline phosphatase placental-like	ALPG	P10696	CHEMBL3402
Phospholipase A-2-activating protein	PLAA	Q9Y263	CHEMBL6114
Monoamine oxidase A	MAOA	P21397	CHEMBL1951
Myoglobin	MB	P02144	CHEMBL2406892
Calcium-activated potassium channel subunit alpha-1	KCNMA1	Q12791	CHEMBL4304
Serine/threonine-protein kinase PIM3	PIM3	Q86V86	CHEMBL5407
Acetylcholinesterase	ACHE	P22303	CHEMBL220
Dual specificity phosphatase Cdc25B	CDC25B	P30305	CHEMBL4804
Arylamine N-acetyltransferase 1	NAT1	P18440	CHEMBL5101

Glycogen synthase kinase-3 beta	GSK3B	P49841	CHEMBL262
Plectin	PLEC	Q15149	CHEMBL1293240
Casein kinase I alpha	CSNK1A1	P48729	CHEMBL2793
Casein kinase I delta	CSNK1D	P48730	CHEMBL2828
Hematopoietic cell protein-tyrosine phosphatase 70Z-PEP	PTPN22	Q9Y2R2	CHEMBL2889
Egl nine homolog 1	EGLN1	Q9GZT9	CHEMBL5697
Arachidonate 5-lipoxygenase	ALOX5	P09917	CHEMBL215
Cyclin-dependent kinase 5/CDK5 activator 1	CDK5R1 CDK5	Q15078 Q00535	CHEMBL1907600
Cyclin-dependent kinase 2/cyclin A	CDK2 CCNA1 CCNA2	P24941 P78396 P20248	CHEMBL2094128
CDK9/cyclin T1	CDK9 CCNT1	P50750 O60563	CHEMBL2111389
Metabotropic glutamate receptor 2 (by homology)	GRM2	Q14416	CHEMBL5137
Gamma-amino-N-butyrate transaminase	ABAT	P80404	CHEMBL2044
Histone chaperone ASF1A	ASF1A	Q9Y294	CHEMBL3392950
MAP kinase-interacting serine/threonine-protein kinase MNK1	MKNK1	Q9BUB5	CHEMBL4718
Bcl2-antagonist of cell death (BAD)	BAD	Q92934	CHEMBL3817
Leukocyte elastase	ELANE	P08246	CHEMBL248
Angiotensin-converting enzyme (by homology)	ACE	P12821	CHEMBL1808
Alkaline phosphatase, tissue-nonspecific isozyme	ALPL	P05186	CHEMBL5979
Vascular cell adhesion protein 1	VCAM1	P19320	CHEMBL3735
Heme oxygenase 1 (by homology)	HMOX1	P09601	CHEMBL2823
Succinate semialdehyde dehydrogenase	ALDH5A1	P51649	CHEMBL1911
Corticotropin releasing factor receptor 1	CRHR1	P34998	CHEMBL1800
Acyl coenzyme A:cholesterol acyltransferase	CES1	P23141	CHEMBL2265
Steroid 5-alpha-reductase 2	SRD5A2	P31213	CHEMBL1856

Carboxylesterase 2	CES2	O00748	CHEMBL3180
Histone deacetylase 5	HDAC5	Q9UQL6	CHEMBL2563
Histone deacetylase 7	HDAC7	Q8WUI4	CHEMBL2716
Hepatocyte growth factor receptor	MET	P08581	CHEMBL3717
Transthyretin	TTR	P02766	CHEMBL3194
Leucine-rich repeat serine/threonine-protein kinase 2	LRRK2	Q5S007	CHEMBL1075104
D-amino-acid oxidase	DAO	P14920	CHEMBL5485
Protein-glutamine gamma-glutamyltransferase	TGM2	P21980	CHEMBL2730
Calmodulin	CALM1	P62158	CHEMBL6093
Matrix metalloproteinase 13	MMP13	P45452	CHEMBL280
Serine/threonine-protein kinase Chk1	CHEK1	O14757	CHEMBL4630
c-Jun N-terminal kinase 1	MAPK8	P45983	CHEMBL2276
c-Jun N-terminal kinase 3	MAPK10	P53779	CHEMBL2637
c-Jun N-terminal kinase 2	MAPK9	P45984	CHEMBL4179
Lysine-specific demethylase 5C	KDM5C	P41229	CHEMBL2163176
Lysine-specific demethylase 4B	KDM4B	O94953	CHEMBL3313832
Lysine-specific demethylase 5B	KDM5B	Q9UGL1	CHEMBL3774295
Lysine-specific demethylase 4A	KDM4A	O75164	CHEMBL5896
Steroid 5-alpha-reductase 1	SRD5A1	P18405	CHEMBL1787
Neuronal acetylcholine receptor; alpha3/beta2	CHRNA3 CHRNB2	P32297 P17787	CHEMBL2109234
Serotonin 1e (5-HT1e) receptor	HTR1E	P28566	CHEMBL2182
Alpha-1d adrenergic receptor	ADRA1D	P25100	CHEMBL223
Alpha-1a adrenergic receptor	ADRA1A	P35348	CHEMBL229
Alpha-1b adrenergic receptor	ADRA1B	P35368	CHEMBL232

GABA receptor alpha-5 subunit (by homology)	GABRA5	P31644	CHEMBL5112
GABA receptor alpha-1 subunit (by homology)	GABRA1	P14867	CHEMBL1962
Retinoic acid receptor gamma	RARG	P13631	CHEMBL2003
Retinoic acid receptor beta	RARB	P10826	CHEMBL2008
Retinoic acid receptor alpha	RARA	P10276	CHEMBL2055
Serum albumin	ALB	P02768	CHEMBL3253
Neprilysin (by homology)	MME	P08473	CHEMBL1944
Induced myeloid leukemia cell differentiation protein Mcl-1	MCL1	Q07820	CHEMBL4361
Apoptosis regulator Bcl-2	BCL2	P10415	CHEMBL4860
Matrix metalloproteinase 3	MMP3	P08254	CHEMBL283
Voltage-gated potassium channel subunit Kv1.3	KCNA3	P22001	CHEMBL4633
Proteasome Macropain subunit MB1	PSMB5	P28074	CHEMBL4662
Matrix metalloproteinase 9	MMP9	P14780	CHEMBL321
Histone deacetylase 9	HDAC9	Q9UKV0	CHEMBL4145
Monoglyceride lipase	MGLL	Q99685	CHEMBL4191
Estrogen receptor alpha	ESR1	P03372	CHEMBL206
Estrogen receptor beta	ESR2	Q92731	CHEMBL242
NADPH oxidase 4	NOX4	Q9NPH5	CHEMBL1250375
N-lysine methyltransferase SETD8	KMT5A	Q9NQR1	CHEMBL1795176
Mannose-6-phosphate isomerase	MPI	P34949	CHEMBL2758
Serotonin 5a (5-HT5a) receptor	HTR5A	P47898	CHEMBL3426
Leukocyte common antigen	PTPRC	P08575	CHEMBL3243
GABA-A receptor; alpha-3/beta-3/gamma-2	GABRB3 GABRA3 GABRG2	P28472 P34903 P18507	CHEMBL2094120

GABA-A receptor; alpha-5/beta-3/gamma-2	GABRB3<br>GABRG2<br>GABRA5	P28472 P18507 P31644	CHEMBL2094122
Tyrosine-protein kinase SRC	SRC	P12931	CHEMBL267
Serine/threonine-protein kinase EEF2K	EEF2K	O00418	CHEMBL5026
Sodium/hydrogen exchanger 1	SLC9A1	P19634	CHEMBL2781
G-protein coupled receptor kinase 2	GRK2	P25098	CHEMBL4079
Group X secretory phospholipase A2	PLA2G10	O15496	CHEMBL4342
Coagulation factor VII/tissue factor	F3	P13726	CHEMBL4081
Glycogen synthase kinase-3 alpha	GSK3A	P49840	CHEMBL2850

Table S2 Prediction Targets of Emodin

Target	Common name	Uniprot ID	ChEMBL ID
Estrogen receptor alpha	ESR1	P03372	CHEMBL206
Serine/threonine-protein kinase PIM1	PIM1	P11309	CHEMBL2147
Estrogen receptor beta	ESR2	Q92731	CHEMBL242
Casein kinase II alpha	CSNK2A1	P68400	CHEMBL3629
Protein-tyrosine phosphatase 4A3	PTP4A3	O75365	CHEMBL4162
Leukocyte elastase	ELANE	P08246	CHEMBL248
Protein farnesyltransferase	FNTA FNTB	P49354 P49356	CHEMBL2094108
Induced myeloid leukemia cell differentiation protein Mcl-1	MCL1	Q07820	CHEMBL4361
Apoptosis regulator Bcl-2	BCL2	P10415	CHEMBL4860
Alpha-ketoglutarate-dependent dioxygenase FTO	FTO	Q9C0B1	CHEMBL2331065
LIM domain kinase 1	LIMK1	P53667	CHEMBL3836
Tyrosine-protein kinase LCK	LCK	P06239	CHEMBL258
Cytochrome P450 19A1	CYP19A1	P11511	CHEMBL1978
P-glycoprotein 1	ABCB1	P08183	CHEMBL4302
Butyrylcholinesterase	BCHE	P06276	CHEMBL1914
Xanthine dehydrogenase	XDH	P47989	CHEMBL1929
Adenosine A3 receptor	ADORA3	P0DMS8	CHEMBL256
Cytochrome P450 1B1	CYP1B1	Q16678	CHEMBL4878
Serine/threonine-protein kinase Aurora-B	AURKB	Q96GD4	CHEMBL2185
Vascular endothelial growth factor receptor 2	KDR	P35968	CHEMBL279
Serine/threonine-protein kinase PLK1	PLK1	P53350	CHEMBL3024
Hepatocyte growth factor receptor	MET	P08581	CHEMBL3717

Tyrosine-protein kinase receptor UFO	AXL	P30530	CHEMBL4895
Epidermal growth factor receptor erbB1	EGFR	P00533	CHEMBL203
Fatty acid synthase	FASN	P49327	CHEMBL4158
Poly [ADP-ribose] polymerase-1	PARP1	P09874	CHEMBL3105
Tankyrase-2	TNKS2	Q9H2K2	CHEMBL6154
Tankyrase-1	TNKS	O95271	CHEMBL6164
Corticotropin releasing factor receptor 1	CRHR1	P34998	CHEMBL1800
Dopamine D3 receptor	DRD3	P35462	CHEMBL234
Aldose reductase	AKR1B1	P15121	CHEMBL1900
Cyclin-dependent kinase 5/CDK5 activator 1	CDK5R1 CDK5	Q15078 Q00535	CHEMBL1907600
Cyclin-dependent kinase 1/cyclin B	CCNB3 CDK1 CCNB1 CCNB2	Q8WWL7 P06493 P14635 O95067	CHEMBL2094127
Cyclin-dependent kinase 6	CDK6	Q00534	CHEMBL2508
ATP-binding cassette sub-family G member 2	ABCG2	Q9UNQ0	CHEMBL5393
Carbonyl reductase [NADPH] 1	CBR1	P16152	CHEMBL5586
Thromboxane-A synthase	TBXAS1	P24557	CHEMBL1835
Maltase-glucoamylase	MGAM	O43451	CHEMBL2074
Serotonin 2c (5-HT2c) receptor	HTR2C	P28335	CHEMBL225
Estrogen-related receptor alpha	ESRRA	P11474	CHEMBL3429
Estrogen-related receptor beta	ESRRB	O95718	CHEMBL3751
L-lactate dehydrogenase A chain	LDHA	P00338	CHEMBL4835
L-lactate dehydrogenase B chain	LDHB	P07195	CHEMBL4940
NADPH oxidase 4	NOX4	Q9NPH5	CHEMBL1250375
Tyrosine-protein kinase receptor FLT3	FLT3	P36888	CHEMBL1974
Tyrosine-protein kinase SYK	SYK	P43405	CHEMBL2599

Glycogen synthase kinase-3 beta	GSK3B	P49841	CHEMBL262
Multidrug resistance-associated protein 1	ABCC1	P33527	CHEMBL3004
Transthyretin	TTR	P02766	CHEMBL3194
Cystic fibrosis transmembrane conductance regulator	CFTR	P13569	CHEMBL4051
Aldo-keto reductase family 1 member B10	AKR1B10	O60218	CHEMBL5983
Interleukin-8 receptor B	CXCR2	P25025	CHEMBL2434
Mannose-6-phosphate isomerase	MPI	P34949	CHEMBL2758
Apoptosis regulator Bcl-X	BCL2L1	Q07817	CHEMBL4625
Insulin-like growth factor binding protein 3	IGFBP3	P17936	CHEMBL3997
Aldehyde dehydrogenase	ALDH2	P05091	CHEMBL1935
Inhibitor of nuclear factor kappa B kinase beta subunit	IKBKB	O14920	CHEMBL1991
Calcium-activated potassium channel subunit alpha-1	KCNMA1	Q12791	CHEMBL4304
Neurotrophic tyrosine kinase receptor type 2	NTRK2	Q16620	CHEMBL4898
Plectin	PLEC	Q15149	CHEMBL1293240
Casein kinase I alpha	CSNK1A1	P48729	CHEMBL2793
Casein kinase I delta	CSNK1D	P48730	CHEMBL2828
Macrophage migration inhibitory factor	MIF	P14174	CHEMBL2085
Bcl2-antagonist of cell death (BAD)	BAD	Q92934	CHEMBL3817
Dual specificity protein phosphatase 3	DUSP3	P51452	CHEMBL2635
Heat shock protein HSP 90-alpha	HSP90AA1	P07900	CHEMBL3880
Adenosine A2a receptor	ADORA2A	P29274	CHEMBL251
6-phosphofructo-2-kinase/fructose-2,6-bisphosphatase 3	PFKFB3	Q16875	CHEMBL2331053
Aryl hydrocarbon receptor	AHR	P35869	CHEMBL3201
Lysine-specific demethylase 4D-like	KDM4E	B2RXH2	CHEMBL1293226

G protein-coupled receptor kinase 6	GRK6	P43250	CHEMBL6144
Isocitrate dehydrogenase [NADP] cytoplasmic	IDH1	O75874	CHEMBL2007625
Receptor-type tyrosine-protein phosphatase S	PTPRS	Q13332	CHEMBL2396508
Cyclin-dependent kinase 2	CDK2	P24941	CHEMBL301
Arginase-1 (by homology)	ARG1	P05089	CHEMBL1075097
Dihydroorotate dehydrogenase (by homology)	DHODH	Q02127	CHEMBL1966
CDC7/DBF4 (Cell division cycle 7-related protein kinase/Activator of S phase kinase)	CDC7	O00311	CHEMBL5443
Protein kinase C delta	PRKCD	Q05655	CHEMBL2996
Protein kinase C epsilon	PRKCE	Q02156	CHEMBL3582
Serine/threonine-protein kinase RAF	RAF1	P04049	CHEMBL1906
Glyoxalase I	GLO1	Q04760	CHEMBL2424
Beta amyloid A4 protein	APP	P05067	CHEMBL2487
Nerve growth factor receptor Trk-A	NTRK1	P04629	CHEMBL2815
Matrix metalloproteinase 9	MMP9	P14780	CHEMBL321
Matrix metalloproteinase 2	MMP2	P08253	CHEMBL333
Matrix metalloproteinase 12	MMP12	P39900	CHEMBL4393
Serine/threonine-protein kinase PIM2	PIM2	Q9P1W9	CHEMBL4523
Lymphocyte differentiation antigen CD38	CD38	P28907	CHEMBL4660
Serine/threonine-protein kinase B-raf	BRAF	P15056	CHEMBL5145
Serine/threonine-protein kinase PIM3	PIM3	Q86V86	CHEMBL5407
DNA topoisomerase I (by homology)	TOP1	P11387	CHEMBL1781
Estradiol 17-beta-dehydrogenase 1	HSD17B1	P14061	CHEMBL3181
Peroxisome proliferator-activated receptor alpha	PPARA	Q07869	CHEMBL239
Peroxisome proliferator-activated receptor gamma	PPARG	P37231	CHEMBL235

Heat shock protein HSP 90-beta	HSP90AB1	P08238	CHEMBL4303
Retinoid X receptor alpha	RXRA	P19793	CHEMBL2061
Hepatocyte nuclear factor 4-alpha	HNF4A	P41235	CHEMBL5398
NAD-dependent deacetylase sirtuin 1	SIRT1	Q96EB6	CHEMBL4506
Protein kinase C eta	PRKCH	P24723	CHEMBL3616
Insulin-like growth factor binding protein 6	IGFBP6	P24592	CHEMBL2139
